# Research is still limited on nutrition and quality of life among older adults

**DOI:** 10.3389/fmed.2023.1225689

**Published:** 2023-09-12

**Authors:** Mary Beth Arensberg, Jaime Gahche, Raquel Clapes, Kirk W. Kerr, Joyce Merkel, Johanna T. Dwyer

**Affiliations:** ^1^Abbott Nutrition Division of Abbott, Columbus, OH, United States; ^2^Office of Dietary Supplements, National Institutes of Health, Bethesda, MD, United States; ^3^Abbott Nutrition Division of Abbott, Granada University Science Park, Granada, Spain; ^4^School of Medicine and Friedman School of Nutrition Science and Policy, Tufts University, Boston, MA, United States

**Keywords:** nutrition, quality of life, healthy aging, older adults, community-living

## Abstract

**Introduction:**

Globally, the number of older adults is growing exponentially. Yet, while living longer, people are not necessarily healthier. Nutrition can positively impact healthy aging and quality of life (QoL). Two decades ago, nutrition and diet were rarely viewed as key QoL domains, were not part of QoL screening, and QoL studies frequently used unvalidated tools. It is unclear how the nutrition and QoL research area may have since evolved.

**Methods:**

A scoping review was conducted in Pubmed of research with community-living older adults (aged ≥65) from developed economies that included 1 of 29 common, valid QoL instruments, nutrition indices, and was published between 1/2000–12/2022. The review followed published methodology guidance and used the Preferred Reporting Items for Systematic Reviews and Meta-analyses (PRISMA) flow diagram to document identified studies and record number of included/excluded studies (based on scoping review’s pre-specified criteria).

**Results:**

Of 258 studies identified initially, 37 fully met scoping review inclusion criteria; only 2 were QoL studies, 30 focused on nutrition, 3 on measurement tool validation/testing, and 2 were other study types. Most studies (*n* = 32) were among populations outside of North America; majority were conducted in Europe (*n* = 22) where the EuroQol 5 Dimension (Eq5D) was used in >1/2 the studies. Of 5 North American studies, the 36-Item Short Form Survey (SF-36) was most frequently used (*n* = 4). Myriad nutrition indices described various aspects of eating, dietary intake, and nutrition status, making comparability between studies difficult. Studies included several different nutrition questionnaires; Mini Nutritional Assessment (MNA) (*n* = 8) or Mini Nutritional Assessment Short Form (MNA-SF) (*n* = 5) were used most frequently. The most frequent anthropometric measure reported was Body Mass Index (BMI) (*n* = 28). Nutrition-related biochemical indices were reported infrequently (*n* = 8).

**Discussion:**

The paucity of studies over the last two decades suggests research on nutrition and QoL among community-living older adults remains underdeveloped. Valid QoL instruments and nutrition indices are now available. To ensure greater comparability among studies it is important to develop consensus on core indices of QoL and particularly nutrition. Greater agreement on these indices will advance further research to support healthy aging and improve QoL for community-dwelling older adults.

## 1. Introduction

Globally the number of older adults is growing exponentially, spurred by increasing longevity and decreasing fertility rates (1). Indeed, in the year 2020, for the first time in history, the number of people aged 60 and older outnumbered children younger than five years of age in the world. By 2030, one in six people will be aged 60 or older, and by 2050, those aged 60 plus will be double in number compared to today, reaching an estimated 2.1 billion. At the same time, the number of oldest old (those aged 80 or older) will triple to reach 426 million by 2050 (2).

The aging of a population is more economically sustainable when older adults are healthy and continue to remain actively engaged in society (3). Older individuals’ contributions to society depend heavily on their health, and so healthy aging has become a priority for health systems throughout the world (4). However, while many people are living longer, their lives are not necessarily healthier, even in higher-income countries such as the United States (US). All countries face major challenges in dealing with current demographic shifts, although the shifts are particularly pronounced in the US. Americans aged 65 and older represented 16% of the population in 2019, and by 2040 this segment is expected to grow to 22%. During the same timeframe, the number of Americans aged 85 or older is projected to more than double, from 6.6 million to 14.4 million (a 118% increase) (5).

Most older Americans, particularly those who are 75 years and older, are afflicted with multiple chronic conditions and other health-related problems (6). Those with major noncommunicable diseases have earlier and steeper rates of functional decline than their healthier peers (7). Healthy aging involves developing and maintaining the functional abilities that enable well-being and a high quality of life (QoL) in older age, including physical, as well as mental and social functional domains (2). This healthy aging goal is recognized in the US *Healthy People 2030* national health goals that include “reducing health problems and improving quality of life for older adults” (8).

Nutrition is fundamental to helping achieve such national health goals. The 2022 US White House Conference on Hunger, Nutrition, and Health described the vital and often unrecognized role that nutrition plays in helping older adults remain healthy and independent (9). In addition, food and eating are part of the pleasures of life (10) that contribute to physical, mental, and social QoL domains. Older adults themselves regard maintaining functional independence and QoL as of primary importance (11). Yet difficulties with eating, poor diet, and other nutrition issues including malnutrition often remain unidentified, although they are potent contributors to frailty, functional impairments, and poor QoL, especially among the very old. Further, age-associated changes in diet and nutrition status are also frequently involved in the development, severity, and/or exacerbation of many chronic degenerative diseases that have an impact on QoL (12).

There is evidence of a link between nutrition and QoL but there is a dearth of research on this important area of healthy aging (13). Four decades ago, the 1979 US *Surgeon General’s Report on Health Promotion and Disease Prevention* proclaimed that the main goal for older adults was to improve their health and QoL, and recognized nutrition as a factor that could help increase older adults’ independence, self-sufficiency, and QoL (14). Over 20 years later a review of nutrition and QoL in older adults found that nutrition and diet were still not part of mainstream research on QoL and were seldom included among key QoL domains (15). The absence of both nutrition in QoL research and QoL considerations in nutrition research after all those years is puzzling since the connection between the two is so relevant to both health policy and older adults themselves.

In their nutrition and QoL review, Amarantos et al. found that the diversity of QoL screening tools was limited and that unvalidated QoL screening tools were frequently used in studies (15). Siette et al. recently summarized existing research on the validity and reliability of 29 commonly used, self-reported instruments for assessing QoL among older adults (16). However, Siette et al. (16) did not consider whether any of these QoL instruments included nutrition.

To guide the development of further research and inform healthy aging policy, we sought to identify how the intersection between nutrition and QoL studies in community-living older adults has evolved in the last 20 years. Specifically, we conducted a scoping review to determine: (1) how much QoL research in community-living older adults included nutrition indices and used one or more of 29 common, validated QoL instruments and (2) how much research on nutrition in community-living older adults included one or more of the same 29 QoL instruments.

## 2. Methods

We performed a scoping review with the assistance of a scientific and health communications expert (JM) to examine the research literature, focusing on studies with community-living older adults that assessed both nutrition and QoL and used one or more of the 29 common, validated QoL instruments identified in Siette et al. (16). The review followed published guidance on methodology (17). The Preferred Reporting Items for Systematic Reviews and Meta-analyses (PRISMA) flow diagram was used to document the studies we identified and record the number of studies included and excluded based on our pre-specified criteria as detailed below.

### 2.1. Search strategy

Studies were included if they had: (1) one or more of the 29 common, validated general QoL instruments used in their entirety, (2) a measure of nutrition status and/or a nutrition intervention, (3) a research population of older adults aged 65 and over who were living in the community or independently, and (5) a study population in a country that has a developed economy. Search terms were a combination of nutrition terms (i.e., nutrition, nutritional, food, diet, hunger, food insecurity) AND independent or community living AND the QoL instruments specified in the paper by Siette et al. AND older adults (complete research search string available in the Supplementary Figure). Articles were retrieved from Pubmed and included those published between January 1, 2000, and December 31, 2022.

Studies were also identified through citations from relevant literature reviews that were initially included in the Pubmed retrieval. Specifically, all systematic reviews, meta-analyses and umbrella reviews were ultimately excluded from our final list of studies but were first screened to identify any studies in the reviews that met our pre-specified inclusion criteria but were not found in the Pubmed search.

### 2.2. Study selection and data extraction

#### 2.2.1. Pre-specified inclusions

At least one of the 29 general QoL instruments that Siette et al. recently reviewed for validity and reliability (16) had to be used in included studies. These instruments were: Alzheimer’s Disease-Related Quality of Life (ADRQOL), Assessment of Quality of Life instrument (including AQoL-8, AQoL-4D, AQoL-6D, AQoL-7D AQoL-8D versions), Adult Social Care Outcomes Toolkit (ASCOT), Comfort Around Dying-End of Life in Dementia (CAD-EOLD), Comprehensive Quality of Life Scale (COMQOL), 15-Dimensional instrument (15-D), Dementia Quality of Life measure (DEMQOL), Dementia Quality of Life Instrument (DQOL), Duke Health Profile (DUKE), EuroQoL-5 Dimensions (EQ-5D), Health Utility Index (HUI), ICEpop CAPability measure for Older people (ICECAP-O), inter Resident Assessment Instrument Long Term Care Facility (inteRAI (LTCF)), Joy-of-Life Scale (JoLS), Long Term Care Quality Of Life assessment scale (LTC-QOL), Manchester Short Assessment of quality of life (MANSA), Nottingham Health Profile (NHP), Nursing Home Vision-Targeted Health-related QoL (NHVQOL), Oral Health Impact Profile (OHIP), Older Peoples Quality Of Life (OPQOL), Philadelphia Geriatric Centre Moral Scale (PGCMS), Quality of Life in Alzheimer’s Disease (QoL-AD), Quality of Life In Late-Stage Dementia (QUALID), Dementia Specific Quality of Life Instrument (QUALIDEM), Short Form-8 Health Survey (SF-8)/ 12-Item Short Form Survey (SF-12)/36-Item Short Form Survey (SF-36), Satisfaction With Life Scale (SWLS), World Health Organization Quality of Life Scale – AGE (WHOQOL-AGE), WHO Quality of Life-Bref (WHOQOL-BREF), World Health Organization Quality of Life Scale–OLD (WHOQOL-OLD). Note that all of these instruments were described as more general QoL instruments and *not* chronic-disease or nutrition-specific instruments. We also searched for additional well-validated and reliable QoL instruments that might have been developed after the publication of the Siette et al. paper (16); none were identified through our further search.

#### 2.2.2. Pre-specified exclusions

Studies were excluded if the research was: (1) only an abstract, poster, study protocol/design (i.e., no published paper with results), (2) targeted toward a palliative care population, (3) focused on QoL for families/caregivers vs. older adults themselves, (4) using only a portion of a validated QoL instrument vs. the complete instrument, (5) conducted with older adults in assisted living or hospitalized older adults but had no follow-up of these subjects in a community-living setting, (6) published before the year 2000, (7) published in languages other than English, and/or (8) not conducted in a country identified by the United Nations as a “developed economy” (18).

#### 2.2.3. Study selection

Five independent researchers (MBA, JG, RC, KWK, JTD) initially screened the titles and corresponding abstracts identified during the search. Twenty percent of the titles and corresponding abstracts were screened by two researchers. All discrepancies involving whether studies would advance to the next step in the review process were discussed as a group and adjudicated accordingly. Next, the full-text articles were reviewed to confirm that the studies fully met the defined inclusion criteria. All five researchers screened the full-text articles, with 32% of the articles screened by two researchers. Again, all discrepancies regarding whether to include studies in the final review were discussed among the group and adjudicated.

#### 2.2.4. Data extraction

Once the final group of studies was identified, all five researchers were assigned full-text articles to review and more detailed study specifics were extracted. Thirty-two percent of the articles were reviewed by two researchers and all discrepancies in the study specifics extracted were adjudicated as a group. Study-specific details extracted included (1) study focus (nutrition intervention, nutrition status, validation or test of an instrument, quality of life, other), (2) objective of the study, (3) validated QoL instrument used, (4) category of nutrition indices used (i.e., questionnaire, anthropometric, biochemical), (5) description of nutrition indices used, (6) country where the study was conducted, and (7) study title and year published. All data were extracted and entered in a Microsoft Excel worksheet.

## 3. Results

**Figure 1:**
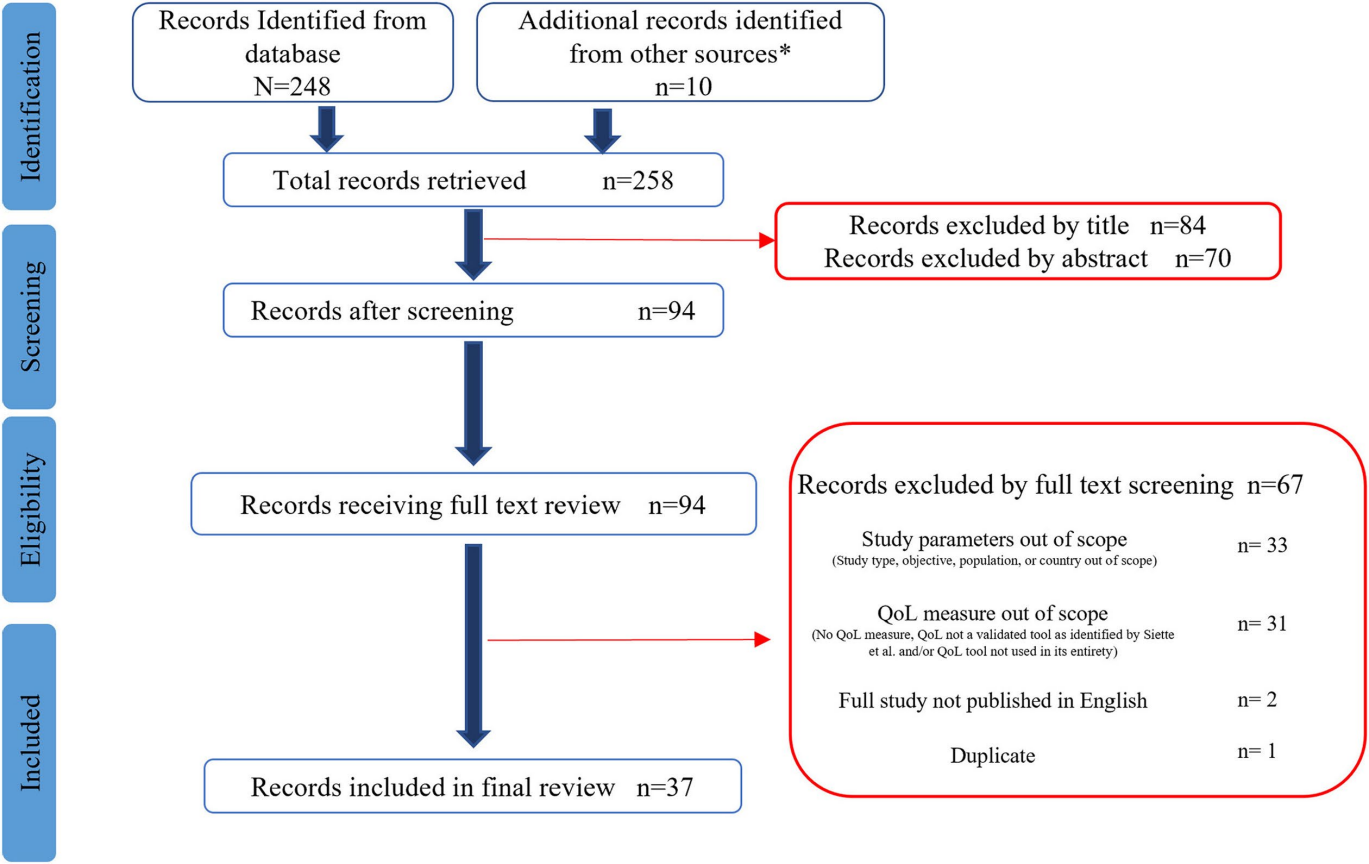
Preferred reporting items for systematic reviews and meta-analyses (PRISMA) flow diagram for nutrition and quality of life scoping review. *Additional articles identified from systematic reviews (19).

Figure 1 illustrates the PRISMA diagram summarizing the number of articles retrieved and screened as well as reasons for exclusions. The Pubmed database search identified 248 articles. An additional 10 articles were identified by screening articles included in systematic reviews, meta-analyses, and umbrella reviews. After all exclusions, 37 articles were included in the final review to identify articles exhibiting the integration of nutrition and QoL research over the last 20 years.

**Figure 2:**
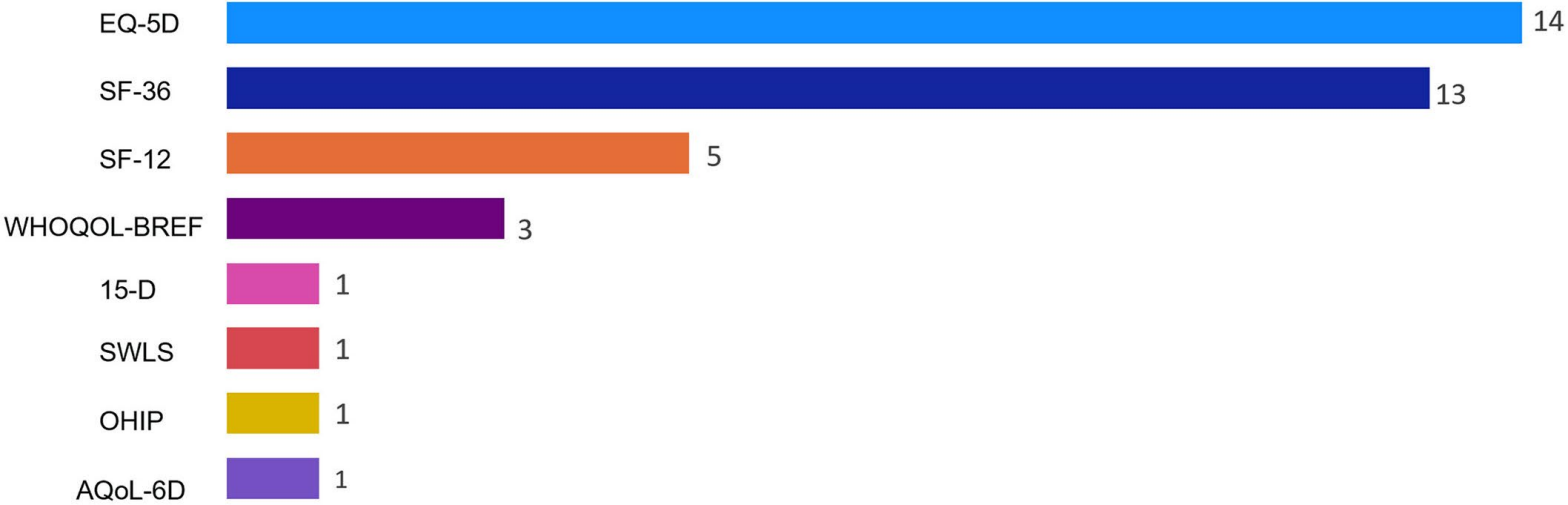
Frequency of quality of life instruments used for studies included in nutrition and quality of life scoping review (*N* = 37). Reported frequencies >37 because some studies used multiple quality of life instruments. 15-D, 15-Dimensional instrument; AQoL-6D, Assessment of Quality of Life instrument-6D Version; EQ-5D, EuroQoL 5-Dimensions; OHIP, Oral Health Impact Profile; SF-12, 12-Item Short Form Health Survey; SF-36, 36-Item Short Form Health Survey; SWLS, Satisfaction With Life Scale; WHOQOL-BREF, World Health Organization Quality of Life-Bref.

**Figure 3:**
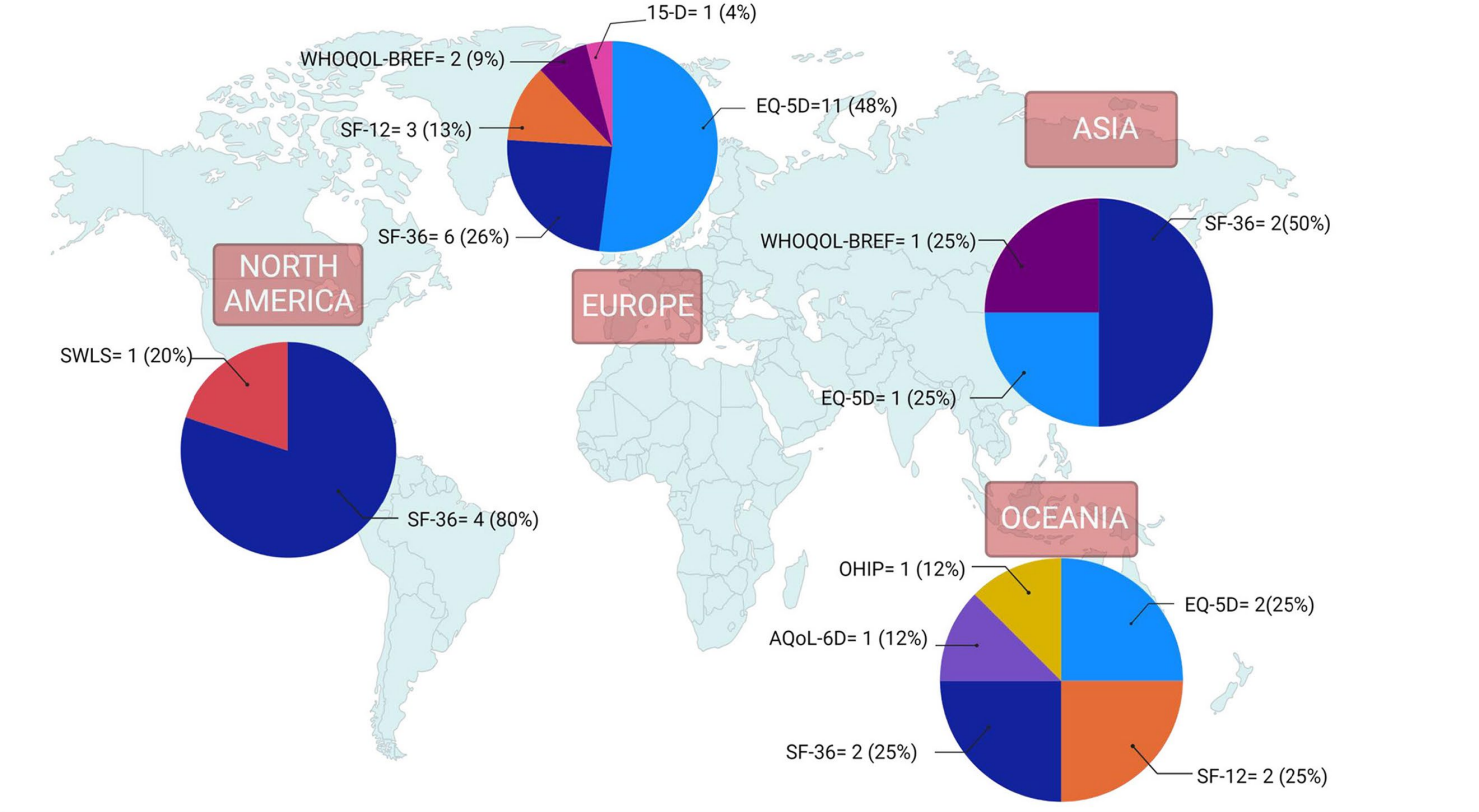
Quality of life instruments by world region for studies included in nutrition and quality of life scoping review (*N* = 37). Reported frequencies >37 because some studies used multiple quality of life instruments and one study included countries from 2 regions. 15-D, 15-Dimensional instrument; AQoL-6D, Assessment of Quality of Life instrument-6D Version; EQ-5D, EuroQoL 5-Dimensions; OHIP, Oral Health Impact Profile; SF-12, 12-Item Short Form Health Survey; SF-36, 36-Item Short Form Health Survey; SWLS, Satisfaction With Life Scale; WHOQOL-BREF, World Health Organization Quality of Life-Bref.

The Supplementary material Table contains study-specific data extracted from the research articles. Of the 37 studies included, the most frequently used QoL instruments were the EQ-5D (*n* = 14), SF-36 (*n* = 13), and SF-12 (*n* = 5) (Figure 2). The WHOQOL-BREF (*n* = 3) was among the QoL instruments that were used less frequently. The 15-D, SWLS, OHIP, and the AQol-6D were each used in only a single study. Most of the studies included in the final review were conducted in Europe (*n* = 22) or Oceania (*n* = 7). The QoL tools they used most frequently also varied by country. Studies conducted in Europe tended to use the EQ-5D, while most studies in North America used the SF-36 (Figure 3). However, it should be noted that North America was represented in only five of the 37 studies included in the final review.

**Figure 4:**
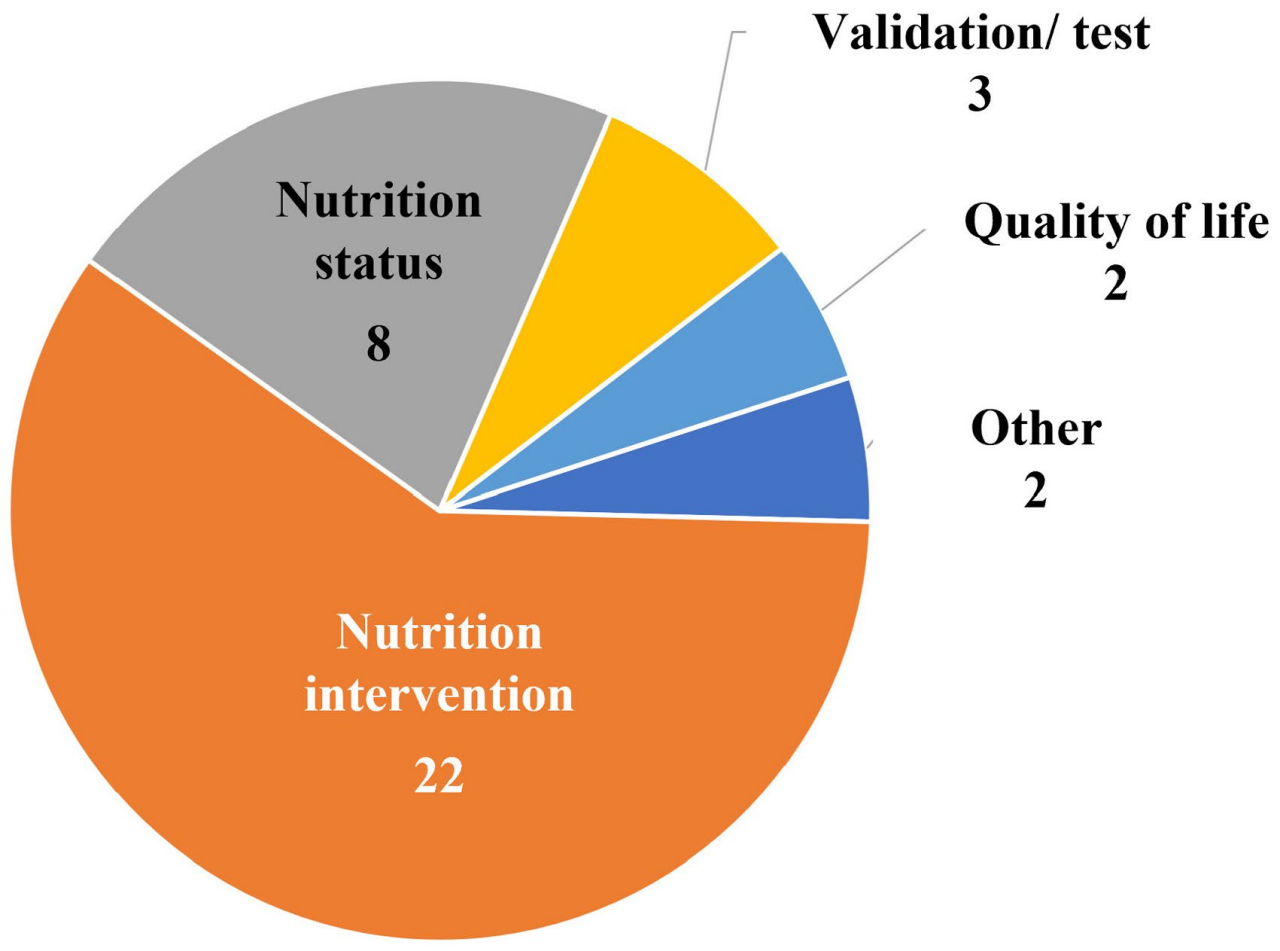
Focus of studies included in nutrition and quality of life scoping review (*N* = 37).

Figure 4 shows that two of the 37 studies included in the final review were focused specifically on QoL. Thirty of the 37 studies were nutrition-focused research studies (describing either nutrition interventions (*n* = 22) or nutrition status (*n* = 8)). The remaining categories of studies were validation and/or testing studies of questionnaires (*n* = 3) and an “other” group (*n* = 2) in which one study concerned healthcare resource use and another involved factors related to frailty.

**Figure 5:**
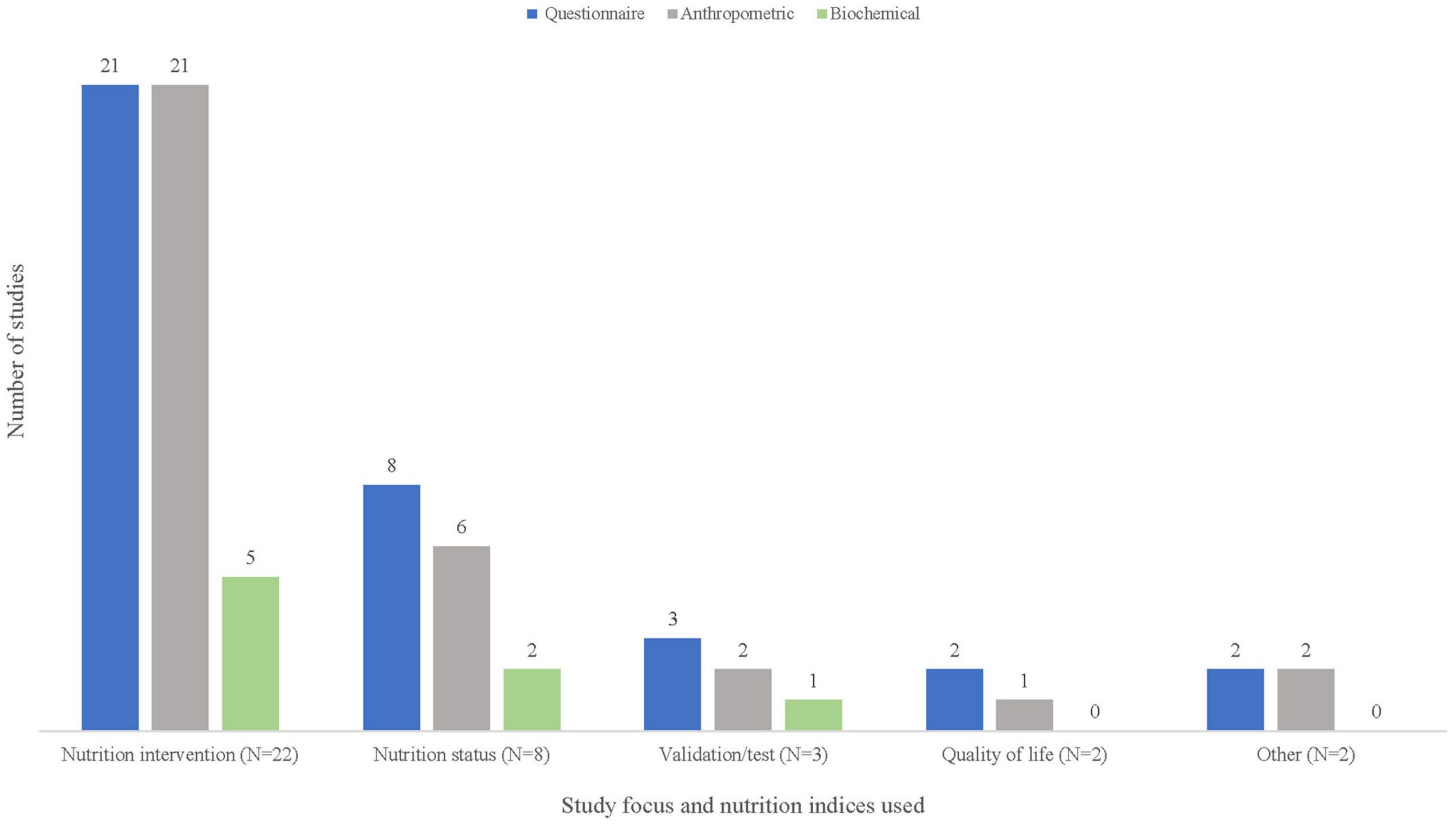
Frequency of nutrition indices used and focus for studies included in nutrition and quality of life scoping review (*N* = 37).

Various nutrition indices were used to evaluate dietary or nutrition status (supplementary material Table). Twenty studies reported collecting some type of dietary intake information. Most of the studies measured nutrition status by questionnaires rather than by biochemical examination (Figure 5). The most commonly used nutrition questionnaire to measure nutrition status was the Mini Nutritional Assessment or MNA (*n* = 8) or its shorter version, the Mini Nutritional Assessment Short-Form or MNA-SF (*n* = 5) (Supplementary Table).

Anthropometric measures (i.e., body mass index (BMI), muscle circumference) were reported in nearly all the nutrition intervention studies but less frequently in studies focused on nutrition status, QoL, or validation/test development studies (Figure 5). BMI was the most collected anthropometric measure and was specifically reported in 28 studies and handgrip strength was the most-commonly reported measure of muscle status (supplementary material Table). Regardless of the type of study, biochemical measures were infrequently reported. Biochemical measures were only collected in 8 of the studies, with albumin being the most commonly reported assay (*n* = 6) (Supplementary Table).

## 4. Discussion

This scoping review identified nutrition and QoL research published during the last two decades that both focused on community-living older adults and included nutrition parameters as well as one or more of 29 common, validated QoL instruments. The number of studies found was limited (*n* = 37). The majority were nutrition intervention (*n* = 22) or nutrition status studies (*n* = 8), while only two were QoL studies. The remainder were either focused on validation/test development (*n* = 3) or other types of studies (*n* = 2).

Across the studies identified, the most-used QoL instruments were the EQ-5D and SF-36, although there were some regional differences; European studies most frequently used the Eq-5D and North American studies mostly used the SF-36. This is not surprising given the origins of these instruments. The EQ-5D is a widely used generic instrument for describing and valuing health, developed by the EuroQol Group. It is a preference-based instrument, with one question for each of five dimensions: mobility, self-care, usual activities, pain/discomfort, and anxiety/depression (20). The individual questions have a range of three or five responses, depending on the instrument version (3 L or 5 L).

The SF-36 is an older, longer instrument first developed in the US at the Rand Corporation. It assesses eight health concepts: physical functioning, role limitations due to physical problems, social functioning, bodily pain, general mental health, role limitations due to emotional problems, vitality, and general health perceptions (21). The standard form of the instrument asks for participants to reply to questions according to how they have felt over the previous week and uses Likert-type scales, some with five or six points and others with two or three points. The SF-36 has also been widely used, has excellent psychometrics, (22, 23) and has shorter versions such as the SF-12 (24).

Most of the QoL instruments are self-report questionnaires designed for administration in surveys. Self-report is generally preferred for QoL because there can be significant differences between self-reports and proxy reports, even for people with disabilities (25). Most of the studies that included nutrition indices also used nutrition questionnaires, as might be expected since questionnaires are likely the most feasible tools for use in community settings.

In contrast to the finding that a few common, validated QoL instruments were used in many studies, the studies used myriad nutrition indices that considered different dimensions of the term, including eating and swallowing, food intake, anthropometric, and biochemical measures. Several studies in our review used nutrition questionnaires, most commonly the MNA (*n* = 8) or MNA-SF (*n* = 5). Both the MNA and MNA-SF are widely used (26) and have been validated for use with older adults (27, 28). The original MNA (now described as the “full” MNA) was designed as an 18-item questionnaire to be completed by a healthcare professional. It includes questions about food intake, weight loss, mobility, psychological stress/acute disease, neuropsychological problems, BMI, living arrangement, prescription drug use, pressure injuries, food consumption, and anthropometric measures (mid-arm muscle and calf circumference) and takes an estimated 10–15 min to complete (27). The MNA-SF has six items, with questions on food intake, weight loss, mobility, psychological stress/acute disease, neuropsychological problems, and BMI or calf circumference, and takes 5 min or less to complete (28). A Self-Mini Nutritional Assessment (SELF-MNA) has also been developed and validated (29) although the SELF-MNA was not used in any of the studies included in our review.

Three-quarters of the studies included anthropometric indices such as BMI, which was specifically reported in 28 studies, and they were much more common than biochemical indices which were only listed in 8 studies. This finding was expected because biochemical tests are difficult to collect in community-living populations, the measurements are costly, time-consuming, and when used by themselves without dietary and other information they are not reliable indicators of nutrition status in older adults (30).

### 4.1. Progress and gaps

Patient-reported outcomes are increasingly recognized as important for shared decision making, guidelines, and health policy (31). They also provide a better understanding of the factors associated with patient satisfaction and quality (32). It was heartening to note the progress made in developing and validating QoL instruments, as revealed in Siette et al.’s seminal review summarizing 29 common, validated QoL instruments (16). Validated QoL instruments are generally self-reported and questionnaire-based and thus are easy to use since they do not require physical instruments, biological measurements, or detailed training to complete. The array of instruments available today provides investigators with multiple options that are appropriate and ready for use in nutrition and other studies of factors that can impact the QoL of older adults in the community.

The recommendation made over two decades ago that more studies relating nutrition to quality of life were needed to “illustrate and strengthen claims that nutrition improves quality of life” did not appear to be heeded (15). It is puzzling why there is still such a striking lack of nutrition research in this area, particularly related to nutrition status. Between the years 2000–2022, we found only 30 nutrition research papers that investigated nutrition and/or diet among community-living older adults and included a validated general QoL instrument. Most of these studies were describing the outcomes of nutrition interventions (*n* = 22) rather than describing nutrition status (*n* = 8). Of the nutrition research papers identified, less than a third (*n* = 10) had a primary focus on QoL (i.e., where QoL was included in the study title). Thus, it appears that QoL instruments are still not yet being used routinely in nutrition studies to monitor outcomes among older adults in community settings. QoL is a complex concept that is interpreted and defined differently within and between various health disciplines (33). It may be that because of the number of instruments available to assess QoL, there was confusion on the part of nutrition researchers as to what instruments were most appropriate for nutrition research and thus QoL outcomes were not readily assessed.

The nutrition measures in the reviewed studies were heterogeneous. Specifically, a set of consistent core nutrition measurements was absent, making the comparison of nutrition results between studies difficult. There could be several reasons for this. One is that our review of research over the last 20 years indicated that nutrition indices have evolved over that time. For example, serum albumin is no longer among the criteria used to identify malnutrition (34). Additionally, even today consensus is still lacking among nutrition researchers on a valid and reliable set of core measures for assessing nutrition and nutrition status in all its dimensions, particularly for older adults living in the community (35). In a clinical setting, the nutrition indices generally considered are those related to the five domains of nutrition assessment: (1) food or nutrition-related history, (2) biochemical data, medical tests, and procedures, (3) anthropometric measurements, (4) nutrition-focused clinical and physical findings, and (5) client history (36). Some of these more complete assessment measures may not have been included in the studies we reviewed because the studies’ goals were to describe only a specific aspect of nutrition, or the measures were unfeasible/less readily available in community-living populations than might be the case in clinical settings. It is also possible that in countries without a national health program (such as the US), researchers may have been disinclined to characterize all the complexities of nutrition status when they lacked the ability/means to fully ameliorate identified problems.

Lack of clarity on what constitutes appropriate nutrition status measures may have contributed to the very limited number of QoL studies (*n* = 2) we identified that included nutrition measures. It may also be the reason why only one study had a primary focus on nutrition, as was indicated in the study title. Interestingly, a recent systematic review of QoL research in medicine and health sciences did not identify any studies on nutrition and QoL, although that research review was limited to one random week’s “snapshot” because researchers were concerned about the high number of QoL articles published annually (33) and thus their timeframe differed markedly from our review of 22 years of published research.

It is disheartening that given the continued, growing emphasis on patient-centered care (37) and healthy aging (4) there is still such a gap in nutrition and QoL research and the number of studies is so limited. Many older adults have one or more chronic diseases (6) and research on chronic disease, such as primary prevention studies like PREDIMED (38), points to the importance of including nutrition in multidimensional approaches to impact health outcomes (39). However, traditional indicators such as reduced morbidity may be less meaningful for older adults themselves than subjectively assessed symptomatic improvements that may relate to their QoL (15). Indeed, defining QoL may allow healthcare providers to shift from minimizing individuals’ disabilities toward maximizing their abilities (40). Several age-associated nutrition changes can also impact QoL, from decreased intake to alteration in nutrient needs, and these changes could benefit from targeted nutrition interventions if the nexus between those changes and QoL could be better elucidated (15). Further, a number of older adults face food insecurity and that can impact QoL as well (41). Such social risks for poor quality of life must be identified before the risks can be addressed by the provision of appropriate health and social services. Those conducting community-based research may not be prepared or able to do so, and this could be another reason for the limited research on nutrition and QoL. However, it is incumbent upon health professionals to investigate the scope of such problems if societal action is necessary.

An additional gap at the nexus of nutrition and QoL is the potential lack of a domain specifically involving food, diet, and eating in QoL instruments. Investigation of whether the 29 common, validated QoL instruments explicitly included food, diet, or eating-related questions was beyond the range of our scoping review. However, these are important factors in the enjoyment of life and for sustenance (42). Older adults with higher diet quality have been found to have higher QoL (43). We did identify one study in our review that also included the Satisfaction with Food-Related Life scale (44). That scale has seven questions related to the positives, negatives, pleasure, and satisfaction of food and meals and has been validated among older adults (42). Other QoL scales specific to nutrition also exist, including the Nutrition Quality of Life Survey (45), Quality of Life Factors Questionnaire (46), and an instrument for measuring QoL related to nutrition in the general population (47); but in the studies we identified none of these instruments were used.

### 4.2. Opportunities and implications for research and policy

There are many challenges involved in promoting independence and healthy aging in an increasingly older population. Chief among them are: determining how important factors like nutrition impact QoL, identifying actionable interventions through research, and then implementing policies that follow up on the findings. The 2022 White House Conference on Hunger, Nutrition, and Health underscored the need for intervention, research, and education on nutrition and healthy aging (9). Yet, as was evident in the limited body of research that we found, risk factors for poor QoL related to nutrition for older adults living in the community is still a gap area. This could be exacerbated because there may be differing requirements and tools for nutrition screening across various countries and populations even as there is consensus on the diagnostic criteria for malnutrition, such as the Global Leadership Initiative on Nutrition (GLIM) criteria (48). Further, even when nutrition and QoL were considered in one setting, research rarely tracked if or how nutrition status or QoL changed as older adults traversed the health and social services care chain. Only one study in our review specifically investigated care transitions and patients’ hospital-to-home journeys. For those older adults who are screened and identified as positive for poor nutrition and/or decreased QoL and who have remediable problems, there is an unmet need for additional assessment, effective interventions, and continuing surveillance. Guidance documents such as those from the World Health Organization (49) and the National Academies of Sciences, Engineering, and Medicine (50) provide strategies for doing so.

Additional opportunities for future research include the need for greater collaboration between nutrition and QoL experts to ensure that appropriate instruments and indices in both areas of research are included in study design and implementation. Maintenance of QoL is one of the most important outcomes of care services for older adults (51) and yet there is a lack of nutrition and QoL studies, particularly in North America. Nutrition researchers in this region should include validated QoL measurements more frequently in their studies of community-living older adults. The use of a consistent QoL instrument such as the SF-36 or SF-12 in North America would permit greater comparability of the results between research investigations. Overall, greater consensus among nutrition researchers on standardized, validated nutrition status core measures for community-dwelling populations (specifically on questionnaires and anthropometric measurements) could make it easier for QoL researchers to include appropriate nutrition measures and thus lead to a more complete picture of nutrition’s impact on QoL in older adults.

### 4.3. Strengths and limitations

This review had several strengths. It updates the work of Amarantos et al. (15) and adds a nutrition perspective to the seminal work of Siette et al. (16). Also, it is one of the few studies that has considered the nexus between nutrition and QoL and identified several gaps that are important to address in considering the role of nutrition in healthy aging research and policy. Its limitations include that it focused only on community-living older adult populations in developed economies and specifically searched for research including at least one of 29 common, validated general QoL instruments used in their entirety. There is a need to consider the body of literature for additional care settings--assisted living facilities, nursing homes, and acute care hospitals--and for other countries beyond developed economies. Studies of nutrition using condition-specific QoL measurements developed for older adults with a particular disease or condition were not included in our review, and these deserve attention as well. In addition, future research could review other validated QoL instruments such as those that are nutrition-specific.

## 5. Conclusion

Autonomy and living at home are valued by older adults. Healthy aging and a high QoL are critical to achieving these goals. Nutrition is a fundamental and potentially modifiable factor whose influence is often ignored as a contributor to healthy aging and QoL outcomes and to date the research area on nutrition and QoL remains underdeveloped and neglected. There are valid QoL instruments and nutrition measures that could be incorporated into research among older adult populations, although the lack of consensus on specific indices, particularly for nutrition, is a barrier. It is imperative that agreement be reached on the appropriate instruments and measures to use to identify the most successful screening, assessment, and intervention strategies that ensure healthy aging and QoL of older adults.

## Data availability statement

The original contributions presented in the study are included in the article/Supplementary material, further inquiries can be directed to the corresponding author.

## Author contributions

MA, JG, KK, and JD: conceptualization. JM: research literature search. MA, JG, RC, KK, and JD: writing and reviewing. All authors have read and agreed to the submitted version of the manuscript.

## Conflict of interest

MA and KK were employees and stockholders of Abbott, RC was an intern for Abbott, JD holds stock in several food and drug companies.

The remaining authors declare that the research was conducted in the absence of any commercial or financial relationships that could be construed as a potential conflict of interest.

## Publisher’s note

All claims expressed in this article are solely those of the authors and do not necessarily represent those of their affiliated organizations, or those of the publisher, the editors and the reviewers. Any product that may be evaluated in this article, or claim that may be made by its manufacturer, is not guaranteed or endorsed by the publisher.
