# Supplementary material for: Research is still limited on nutrition and quality of life among older adults
Source: Front Med (Lausanne). 2023 Sep 12;10:1225689. doi: 10.3389/fmed.2023.1225689 (PMC10536330; doi:10.3389/fmed.2023.1225689)
Supplement: Supplementary file 1 [file Table_1.pdf]

Supplementary Material

Figure. Complete research string used for nutrition and quality of life scoping review.

Search String
("nutrition"[Title/Abstract] OR "nutritional"[Title/Abstract]
OR "food"[MeSH Terms] OR "food"[Title/Abstract]
OR "diet"[MeSH Terms]
OR "hunger"[MeSH Terms]
OR "food insecurity"[MeSH Terms])

AND
("Independent Living"[Mesh] OR community living)

AND
("15-D"[All Fields] OR "15-Dimensional instrument"[All Fields] OR "Alzheimer’s Disease-Related Quality of Life"[All Fields] OR "ADRQOL"[All Fields] OR "AQoL-8D"[All Fields] OR "Assessment of Quality of Life instrument – 8D Version" [All Fields] OR "ASCOT"[All Fields] OR "Adult Social Care Outcomes Toolkit" [All Fields] OR "CAD-EOLD"[All Fields] OR " Comfort Around Dying-End of Life in Dementia" [All Fields] OR "COMQOL"[All Fields] OR "Comprehensive Quality of Life Scale" [All Fields] OR "DEMQOL"[All Fields] OR "Dementia Quality of Life measure"[All Fields] OR "DUKE"[All Fields] OR "Duke Health Profile "[All Fields] OR "DQOL"[All Fields] OR "Dementia Quality of Life Instrument"[All Fields] OR "EQ-5D"[All Fields] OR "EuroQol - 5 Dimensions "[All Fields] OR "EuroQol" [All Fields] OR "HUI2/3"[All Fields] OR "Health Utility Index " [All Fields] OR "ICECAP-O"[All Fields] OR "ICEpop CAPability"[All Fields] OR ("inter rai"[All Fields] AND "ltcf"[All Fields]) OR "Inter-RAI (LTCF)"[All Fields] OR "JoLS"[All Fields] OR "Joy-of-Life Scale"[All Fields] OR "MANSA"[All Fields] OR "Manchester Short Assessment"[All Fields] OR "NHP"[All Fields] OR "Nottingham Health Profile"[All Fields] OR "NHVQOL"[All Fields] OR "Nursing Home Vision-related Quality Of Life"[All Fields] OR "OHIP"[All Fields] OR " Oral Health Impact Profile"[All Fields] OR "OPQOL"[All Fields] OR "Older Peoples Quality Of Life"[All Fields] OR "PGCMS"[All Fields] OR "Philadelphia Geriatric Centre Moral Scale "[All Fields] OR "QOL-AD"[All Fields] OR "Quality of Life in Alzheimer’s Disease"[All Fields] OR "LTC-QOL"[All Fields] OR "Long Term Care Quality Of Life assessment"[All Fields] OR "QUALID"[All Fields] OR "Quality of Life In Late-Stage Dementia"[All Fields] OR "QUALIDEM"[All Fields] OR "Dementia Specific Quality of Life Instrument"[All Fields] OR "SF-36"[All Fields] OR "36 Item Shot Form"[All Fields] OR "SWLS"[All Fields] OR "Satisfaction With Life Scale"[All Fields] OR "whoqol age"[All Fields] OR "World Health Organization Quality of Life Scale - AGE"[All Fields] OR "WHOQOL-BREF"[All Fields] OR "World Health Organization Quality of Life Scale - BREF"[All Fields] OR "WHOQOL-OLD"[All Fields] OR "World Health Organization Quality of Life Scale – OLD"[All Fields]
OR
"quality of life"[MeSH Terms])

AND
((2000:2023[pdat]) AND (aged[Filter] OR 80andover[Filter]))

Table. Nutrition indices and QoL instruments used in studies included in nutrition and quality of life scoping review.

Study Objective	QoL Instrument(s) used	Nutrition Indices Used: Questionnaire			Nutrition Indices Used: Anthropometric				Nutrition Indices Used: Biochemical			Country	Title (Year)
		Diet[1]	Difficulties[2]	Nutrition screening tools	Height, weight, and/or weight change	BMI	Skinfold(s) and/or circumference(s)	Bioelectrical impedance, or dual-energy x-ray[3]	Handgrip strength	Plasma vitamins	Serum albumin		
**Nutrition Intervention-focused Studies**													
Examine if nutrition supplementation after discharge from hospital improved nutrition status and functional outcomes, or reduced healthcare costs.	EQ-5D	✓			✓	✓	✓		✓			United Kingdom (UK)	A prospective randomised controlled trial of nutritional supplementation in malnourished elderly in the community: clinical and health economic outcomes (2004) (1)
Examine whether supplementation with multivitamins and multiminerals influenced self-reported days of infection, use of health services and QoL.	EQ-5D, SF-12	✓										Scotland	Effect of multivitamin and multimineral supplements on morbidity from infections in older people (MAVIS trial): pragmatic, randomised, double blind, placebo controlled trial (2005) (2)
Examine effect of individually-adapted nutrition counselling in weight loss among older adults 3 months after discharge from rehabilitation institution.	EQ-5D		✓	NRS-2002	✓	✓						Norway	Effect on body weight, quality of life and appetite following individualized, nutritional counselling to home-living elderly after rehabilitation - an open randomized trial (2017) (3)
Examine efficacy of providing early nutrition intervention and its continuation in older patients discharged from acute care.	EQ-5D			MST, PG-SGA	✓	✓	✓		✓		✓	Australia	Investigation of the benefits of early malnutrition screening with telehealth follow up in elderly acute medical admissions (2017) (4)

Table. *(continued)*

Study Objective	QoL Instrument(s) used	Nutrition Indices Used										Country	Title (Year)
		Questionnaire			Anthropometric					Biochemical			
		Diet[1]	Difficulties[2]	Nutrition screening tools	Height, weight, and/or weight change	BMI	Skinfold(s) and/or circumference(s)	Bioelectrical impedance, or dual-energy x-ray[3]	Handgrip strength	Plasma vitamins	Serum albumin		
Test acceptability, feasibility, preliminary efficacy of technology-supported energy- and protein-enforced home-delivered meals for older patients discharged from hospital.	EQ-5D	✓	✓	NRS-2002	✓	✓			✓			Denmark	Older patients' use of technology for a post-discharge nutritional intervention – A mixed-methods feasibility study (2017) (5)
Evaluate effectiveness of diabetes-specific oral nutrition supplement on nutrition status, QoL, function in malnourished/risk of malnutrition community-dwelling, nursing home-dwelling older adults with type 2 diabetes mellitus.	EQ-5D			MNA	✓	✓						Spain	Effectiveness of an oral diabetes-specific supplement on nutritional status, metabolic control, quality or life, and functional status in elderly patients. A multicentre study (2019) (6)
Understand if older adults have improvement in HRQoL, muscle strength, nutrition status, and other outcomes (including Satisfaction with Food-related Life) when involved in own meals as part of a rehabilitation program.	EQ-5D	✓	✓		✓	✓						Denmark	Involving community-dwelling older adults in activities related to meals as part of a rehabilitation program: a single-blinded cluster-controlled study (2019) (7)

Table. *(continued)*

Study Objective	QoL Instrument(s) used	Nutrition Indices Used										Country	Title (Year)
		Questionnaire			Anthropometric					Biochemical			
		Diet[1]	Difficulties[2]	Nutrition screening tools	Height, weight, and/or weight change	BMI	Skinfold(s) and/or circumference(s)	Bioelectrical impedance, or dual-energy x-ray[3]	Handgrip strength	Plasma vitamins	Serum albumin		
Test effectiveness of a resistance exercise and dietary protein intervention for older adults.	EQ-5D	✓	✓	SNAQ	✓	✓		✓				Netherlands	Effectiveness of a diet and resistance exercise intervention on muscle health in older adults: ProMuscle in practice (2020) (8)
Evaluate effects of ONS and dietary advice versus dietary advice alone on intake, weight, QoL, healthcare use, satisfaction in malnourished free-living older people.	EQ-5D	✓		MUST	✓	✓						UK	Ready-made oral nutritional supplements improve nutritional outcomes and reduce health care use--a randomised trial in older malnourished people in primary care (2020) (9)
Evaluate impact of nutrition supplementation on nutrition status, muscle strength, perceived health, functional status in population of community-living, frail, undernourished older adults.	SF-36	✓			✓	✓	✓		✓			Canada	Benefits of nutritional supplementation in free-living, frail, undernourished elderly people: a prospective randomized community trial (2002) (10)
Determine effect of dietary intervention and micronutrient supplementation on self-reported infection.	SF-36	✓			✓	✓	✓			✓		UK	Improvement in nutritional status reduces the clinical impact of infections in older adults (2012) (11)

Table. *(continued)*

Study Objective	QoL Instrument(s) used	Nutrition Indices Used										Country	Title (Year)
			Questionnaire			Anthropometric			Biochemical				
		Diet[1]	Difficulties[2]	Nutrition screening tools	Height, weight, and/or weight change	BMI	Skinfold(s) and/or circumference(s)	Bioelectrical impedance, or dual-energy x-ray[3]	Handgrip strength	Plasma vitamins	Serum albumin		
Examine whether 12-week combined physical exercise training and nutrition intervention improved physical performance, enhanced HRQoL among prefrail older, community-living women.	SF-36	✓						✓	✓		✓	Japan	Effects of a combined physical training and nutrition intervention on physical performance and health-related quality of life in prefrail older women living in the community: a randomized controlled trial (2015) (12)
Examine effect of supplementation with coenzyme Q10 and selenium on number of days out of hospital and QoL.	SF-36					✓						Denmark	Improved health-related quality of life, and more days out of hospital with supplementation with selenium and coenzyme Q10 combined. Results from a double blind, placebo-controlled prospective study (2015) (13)
Compare effects of 2 nutrition follow-up interventions in preventing short-term deterioration in ADLs; compare their effects on physical function, emotional health and HRQoL.	SF-36		✓	MNA	✓	✓			✓			Denmark	Early nutritional follow-up after discharge prevents deterioration of ADL functions in malnourished, independent, geriatric patients who live alone - a randomized clinical trial (2016) (14)
Examine effect of oral testosterone and nutrition supplement on hospital admissions.	SF-36	✓		MNA	✓	✓		✓	✓			Australia	The randomized control trial of the effects of testosterone and a nutritional supplement on hospital admissions in undernourished, community dwelling, older people (2016) (15)

Table. *(continued)*

Study Objective	QoL Instrument(s) used	Nutrition Indices Used										Country	Title (Year)
		Questionnaire			Anthropometric					Biochemical			
		Diet[1]	Difficulties[2]	Nutrition screening tools	Height, weight, and/or weight change	BMI	Skinfold(s) and/or circumference(s)	Bioelectrical impedance, or dual-energy x-ray[3]	Handgrip strength	Plasma vitamins	Serum albumin		
Evaluate effects of intervention including nutrition telemonitoring, nutrition education, nurse follow-up on nutrition status, diet quality, appetite, physical functioning, QoL.	SF-36	✓	✓	MNA-SF, DHD-FFQ	✓	✓						Netherlands	Effects of a multi-component nutritional telemonitoring intervention on nutritional status, diet quality, physical functioning and quality of life of community-dwelling older adults (2018) (16)
Examine effects of physical activity program in combination with protein supplementation on HRQoL, depressive symptoms in community-dwelling, mobility-limited older adults.	SF-36			MNA-SF		✓		✓	✓	✓		Sweden and United States (US)	Effect of exercise and nutritional supplementation on health-related quality of life and mood in older adults: the VIVE2 randomized controlled trial (2018) (17)
Consider LBM, muscle performance, physical function, protein intake needed in older adults with physical function limitation to optimize anabolic response to testosterone.	SF-36	✓			✓	✓		✓			✓	US	Effect of protein intake on lean body mass in functionally limited older men: a randomized clinical trial (2018) (18)
Evaluate effectiveness of exercise program and 2 types of protein supplements in pre-frail community-living older adults.	SF-36	✓		MNA-SF				✓	✓			Australia	A randomized controlled pilot exercise and protein effectiveness supplementation study (EXPRESS) on reducing frailty risk in community-dwelling older people (2021) (19)

Table. *(continued)*

Study Objective	QoL Instrument(s) used	Nutrition Indices Used										Country	Title (Year)
			Questionnaire			Anthropometric				Biochemical			
		Diet[1]	Difficulties[2]	Nutrition screening tools	Height, weight, and/or weight change	BMI	Skinfold(s) and/or circumference(s)	Bioelectrical impedance, or dual-energy x-ray[3]	Handgrip strength	Plasma vitamins	Serum albumin		
Evaluate effectiveness of interdisciplinary multicomponent intervention program to prevent disability in older people.	SF-12	✓		SNAQ					✓			Netherlands	The effectiveness of a PRoactive Multicomponent Intervention Program on disability in independently living older people: a randomized controlled trial (2018) (20)
Examine effect of tailored nutrition guidance on nutrition, HRQoL, falls in people with Alzheimer's disease.	15-D	✓		MNA	✓	✓						Finland	Nutritional guidance improves nutrient intake and quality of life, and may prevent falls in aged persons with Alzheimer disease living with a spouse (NuAD Trial) (2015) (21)
Evaluate effectiveness of introducing dietitian-led discharge planning and follow-up program within usual resources to improve nutrition, functional recovery in older adults at risk of malnutrition.	AQoL-6D			MST, MNA	✓	✓			✓			Australia	Improving nutritional discharge planning and follow up in older medical inpatients: Hospital to Home Outreach for Malnourished Elders (2018) (22)

Table. *(continued)*

Study Objective	QoL Instrument(s) used	Nutrition Indices Used										Country	Title (Year)
		Questionnaire			Anthropometric					Biochemical			
		Diet[1]	Difficulties[2]	Nutrition screening tools	Height, weight, and/or weight change	BMI	Skinfold(s) and/or circumference(s)	Bioelectrical impedance, or dual-energy x-ray[3]	Handgrip strength	Plasma vitamins	Serum albumin		
**Nutrition Status-focused Studies**													
Evaluate QoL of noninstitutionalized population > 75 years old by determining nutrition and health status; investigate relationship between nutrition risk and QoL.	EQ-5D			MNA		✓						Spain	Quality of life and risk of malnutrition in a home-dwelling population over 75 years old (2017) (23)
Evaluate association between oral/general health, QoL, malnutrition.	EQ-5D		✓		✓	✓						Netherlands	Are edentulousness, oral health problems and poor health-related quality of life associated with malnutrition in community-dwelling elderly (Aged 75 Years and Over)? A cross-sectional study (2018) (24)
Evaluate association between QoL, mortality, nutrition status, ADLs.	SF-36		✓								✓	Japan	Impact of the serum level of albumin and self-assessed chewing ability on mortality, QOL, and ADLs for community-dwelling older adults at the age of 85: A 15 year follow up study (2020) (25)
Evaluate association between preoperative nutritional status and preoperative physical function, patient reported QoL, body composition in colorectal cancer patients awaiting elective surgery.	SF-36			PG-SGA	✓	✓		✓	✓		✓	Canada	Colorectal cancer patients with malnutrition suffer poor physical and mental health before surgery (2021) (26)

Table. *(continued)*

Study Objective	QoL Instrument(s) used	Nutrition Indices Used										Country	Title (Year)
		Questionnaire			Anthropometric					Biochemical			
		Diet[1]	Difficulties[2]	Nutrition screening tools	Height, weight, and/or weight change	BMI	Skinfold(s) and/or circumference(s)	Bioelectrical impedance, or dual-energy x-ray[3]	Handgrip strength	Plasma vitamins	Serum albumin		
Establish prevalence of high nutrition risk and associated health and social risk factors for New Zealand Māori and non-Māori in advanced age.	SF-12			SCREEN II					✓			New Zealand	Health and social factors associated with nutrition risk: results from Life and Living in Advanced Age: A cohort study in New Zealand (LILACS NZ) (2015) (27)
Evaluate association of undernutrition risk with HRQoL, life satisfaction controlling for age, gender, marital status, economic status, housing arrangement, education level, functional ability, and diseases.	SF-12	✓	✓		✓	✓	✓					Sweden	Prevalence and association of undernutrition with quality of life among Swedish people Aged 60 years and above: results of the SNAC-B study (2015) (28)
Determine 4-year outcomes of community-living older adults identified at 'nutrition risk' in 2014 Health, Work and Retirement Study.	SF-12			SCREEN II-AB								New Zealand	Malnutrition Risk: Four Year Outcomes from the Health, Work and Retirement Study 2014 to 2018 (2022) (29)

Table. *(continued)*

Study Objective	QoL Instrument(s) used	Nutrition Indices Used: Questionnaire			Anthropometric					Biochemical		Country	Title (Year)
		Diet[1]	Difficulties[2]	Nutrition screening tools	Height, weight, and/or weight change	BMI	Skinfold(s) and/or circumference(s)	Bioelectrical impedance, or dual-energy x-ray[3]	Handgrip strength	Plasma vitamins	Serum albumin		
Examine relationship between socio-demographic factors, social resources, functional status, QoL, malnutrition/malnutrition risk in older adults.	WHOQOL-BREF			MNA-SF	✓	✓						Spain	Quality of life, functional impairment and social factors as determinants of nutritional status in older adults: The VERISAÚDE study (2018) (30)
**Validation/Test-focused Studies**													
Examine whether DASC-8 is useful for screening frailty and as comprehensive geriatric assessment.	EQ-5D			MNA-SF		✓			✓		✓	Japan	Use of Dementia Assessment Sheet for Community-based Integrated Care System 8-items (DASC-8) for the screening of frailty and components of comprehensive geriatric assessment (2020) (31)
Relate MNA and SF-36 in older adult population (compare subjective and general health status measured by MNA and SF-36 in this population).	SF-36			MNA								Sweden	Relationship between MNA and SF-36 in a free-living elderly population aged 70 to 75 (2005) (32)

Table. *(continued)*

Study Objective	QoL Instrument(s) used	Nutrition Indices Used										Country	Title (Year)
		Questionnaire			Anthropometric					Biochemical			
		Diet[1]	Difficulties[2]	Nutrition screening tools	Height, weight, and/or weight change	BMI	Skinfold(s) and/or circumference(s)	Bioelectrical impedance, or dual-energy x-ray[3]	Handgrip strength	Plasma vitamins	Serum albumin		
Consider how DST risk categories differ by demographic, anthro-pometric, cognitive, functional, psychosocial, behavioral variables in older adults.	SWLS	✓			✓	✓						US	Differences in psychosocial and behavioral variables by dietary screening tool risk category in older adults (2018) (33)
**Quality of Life-focused Studies**													
Examine association between oral/general health and QoL.	EQ-5D, OHIP	✓	✓									Australia	Health behaviours and quality of life in independently living South Australians aged 75 years or older (2018) (34)
Assess QoL in chronically-ill older adults and its relationship with parameters concerning nutrition status.	WHOQOL-BREF	✓		MNA		✓						Spain	Nutritional status in chronically-ill elderly patients. Is it related to quality of life? (2014) (35)
**Other Studies**													
Assess effect of ONS prescription on hospitalization risk, healthcare cost.	EQ-5D		✓		✓	✓						France	Compliance to oral nutritional supplementation decreases the risk of hospitalisation in malnourished older adults without extra health care cost: Prospective observational cohort study (2020) (36)

Table. *(continued)*

Study Objective	QoL Instrument(s) used	Nutrition Indices Used: Questionnaire			Anthropometric					Biochemical		Country	Title (Year)
		Diet[1]	Difficulties[2]	Nutrition screening tools	Height, weight, and/or weight change	BMI	Skinfold(s) and/or circumference(s)	Bioelectrical impedance, or dual-energy x-ray[3]	Handgrip strength	Plasma vitamins	Serum albumin		
Evaluate factors associated with frailty (including clinical background, physical/ cognitive function, total physical activity, daily protein intake, QoL, frailty status).	WHOQOL-BREF	✓		BDHQ		✓			✓			Japan	Impact of quality of life on future frailty status of rural Japanese community-dwelling older adults (2022) (37)

1. (e.g. deficiencies, intake, variety)
2. (e.g. appetite, dental, eating alone, swallowing, perceived difficulties)
3. (e.g. lean mass, appendicular lean mass, muscle mass, fat mass)

Abbreviations Used

General: ADLs: Activities of Daily Living; BMI: Body Mass Index; DASC-8: Dementia Assessment Sheet for Community-based Integrated Care System 8-items; HRQoL: Health-related Quality of Life; LBM: Lean Body Mass; ONS: Oral Nutritional Supplement; QoL: Quality of Life.

QoL instruments: 15-D: 15-Dimensional instrument; AQoL-6D: Assessment of Quality of Life instrument-6D Version; EQ-5D: EuroQoL 5-Dimensions; OHIP: Oral Health Impact Profile; SF-12: 12-Item Short Form Health Survey; SF-36: 36-Item Short Form Health Survey; SWLS: Satisfaction With Life Scale; WHOQOL-BREF: World Health Organization Quality of Life-Bref.

Nutrition questionnaires: BDHQ: Brief-type Self-administered Diet History Questionnaire; DHD-FFQ: Dutch Health Diet FFQ; DST: Dietary Screening Tool; MNA: Mini Nutritional Assessment; MNA-SF: Mini Nutritional Assessment-Short Form; MST: Malnutrition Screening Tool; MUST: Malnutrition Universal Screening Tool; NRS-2002: Nutritional Risk Screening 2002; PG-SGA: Scored Patient-Generated Subjective Global Assessment; SCREEN II: Seniors in the community: risk evaluation for eating and nutrition, Version II; SCREEN II-AB: SCREEN-II-Abbreviated; SNAQ: Simplified Nutritional Appetite Questionnaire.

References

1. Edington J, Barnes R, Bryan F, Dupree E, Frost G, Hickson M, et al. A prospective randomized controlled trial of nutritional supplementation in malnourished elderly in the community: clinical and health economic outcomes. *Clin Nutr*. (2004) 23:195–204. doi: 10.1016/S0261-5614(03)00107-9
2. Avenell A, Campbell MK, Cook JA, Hannaford PC, Kilonzo MM, NcNeill G, et al. Effect of multivitamin and multimineral supplements on morbidity from infections in older people (MAVIS trial): pragmatic, randomised, double blind, placebo controlled trial. *BMJ*. (2005) 331:324–9. doi: 10.1136/bmj.331.7512.324
3. Andersson J, Hulander E, Rothenberg E, Iversen P. Effect on body weight, quality of life and appetite following individualized, nutritional counselling to home-living elderly after rehabilitation - an open randomized trial. *J Nutr Health Aging*. (2017) 21:811–8. doi: 10.1007/s12603-016-0825-8
4. Sharma Y, Thompson CH, Kaambwa B, Shahi R, Hakendorf P, Miller M. Investigation of the benefits of early malnutrition screening with telehealth follow up in elderly acute medical admissions. *QJM*. (2017) 110:639–47. doi: 10.1093/qjmed/ hcx095
5. Lindhardt T, Nielsen MH. Older patients' use of technology for a post-discharge nutritional intervention – a mixed-methods feasibility study. *Int J Med Inform*. (2017) 97:312–21. doi: 10.1016/j.ijmedinf.2016.10.017
6. Martin PM, Agudo FR, Medina JAL, Paris AS, Santabalbina FT, Pascual JRD, et al. Effectiveness of an oral diabetes-specific supplement on nutritional status, metabolic control, quality or life, and functional status in elderly patients. A multicentre study. *Clin Nutr*. (2019) 38:1253–61. doi: 10.1016/j.clnu.2018.05.007
7. Husted MM, Beck AM, Ulrikkeholm LK. Involving community-dwelling older adults in activities related to meals as part of a rehabilitation program: a single-blinded cluster-controlled study. *Clin Rehabil.* (2019) 33(7):1185-96. doi: 10.1177/0269215519837742
8. Van Dongen EJI, Haveman-Nies A, Doets EL, Dorhout BG, de Groot LCPGM. Effectiveness of a diet and resistance exercise intervention on muscle health in older adults: ProMuscle in practice. *J Am Med Dir Assoc*. (2020) 21:1065–1072.e3. doi: 10.1016/j.jamda.2019.11.026
9. Smith TR, Cawood AL, Walters ER, Guildford N, Stratton RJ. Ready-made oral nutritional supplements improve nutritional outcomes and reduce health care use--a randomised trial in older malnourished people in primary care. *Nutrients*. (2020) 12:517. doi: 10.3390/nu12020517
10. Payette H, Boutier V, Coulombe C, Gray-Donald K. Benefits of nutritional supplementation in free-living, frail, undernourished elderly people: a prospective randomized community trial. *J Am Diet Assoc*. (2002) 102:1088–95. doi: 10.1016/S0002-8223(02)90245-2
11. Forster SE, Powers HJ, Foulds GA, Flower DJ, Hopkinson K, Parker SG, et al. Improvement in nutritional status reduces the clinical impact of infections in older adults. *J Am Geriatr Soc*. (2012) 60:1645–54. doi: 10.1111/j.1532-5415.2012.04118.x
12. Kwon J, Yoshida Y, Yoshida H, Kim H, Suzuki T, Lee Y. Effects of a combined physical training and nutrition intervention on physical performance and health-related quality of life in prefrail older women living in the community: a randomized controlled trial. *J Am Med Dir Assoc*. (2015) 16:263.e1–8. doi: 10.1016/j.jamda.2014.12.005
13. Johansson P, Dahlstrom O, Dahlstrom U, Alehagen . Improved health-related quality of life, and more days out of hospital with supplementation with selenium and coenzyme Q10 combined. results from a double blind, placebo-controlled prospective study. *J Nutr Health Aging*. 19:870–7. doi: 10.1007/s12603-015-0509-9
14. Pedersen JL, Pedersen PU, Damsgaard EM. Early nutritional follow-up after discharge prevents deterioration of ADL functions in malnourished, independent, geriatric patients who live alone - a randomized clinical trial. *J Nutr Health Aging*. (2016) 20:845–53. doi: 10.1007/s12603-015-0629-2

15. Visvanathan R, Piantadosi C, Lange K, Naganathan V, Hunter P, Cameron ID, et al. The randomized control trial of the effects of testosterone and a nutritional supplement on hospital admissions in undernourished, community dwelling, older people. *J Nutr Health Aging*. (2016) 20:769–79. doi: 10.1007/s12603-016-0689-y
16. Doorn-van Atten M, Haveman-Nies A, van Bakel MM, Fery M, Franco M, de Groot LCPGM, et al. Effects of a multi-component nutritional telemonitoring intervention on nutritional status, diet quality, physical functioning and quality of life of community-dwelling older adults. *Br J Nutr* (2018) 119:1185–1194. doi: 10.1017/S0007114518000843
17. von Berens A, Fielding RA, Gustafsson T, Kirn D, Laussen J, Nydahl M, et al. Effect of exercise and nutritional supplementation on health-related quality of life and mood in older adults: the VIVE2 randomized controlled trial. *BMC Geriatr*. (2018) 18:286. doi: 10.1186/s12877-018-0976-z
18. Bhasin S, Apovian CM, Travison TG, Pencina K, Moore LL, Huang G, et al. Effect of protein intake on lean body mass in functionally limited older men: a randomized clinical trial. *JAMA Intern Med*. (2019) 178:530–41. doi: 10.1001/jamainternmed.2018.0008
19. Jadczak AD, Visvanathan R, Barnard R, Luscombe-Marsh N. A randomized controlled pilot exercise and protein effectiveness supplementation study (EXPRESS) on reducing frailty risk in community-dwelling older people. *J Nutr Gerontol Geriatr*. (2021) 40:26–45. doi: 10.1080/21551197.2021.1886222
20. van Lieshout MRJ, Bleijenberg N, Shuurmans MJ, de Wit NJ. The effectiveness of a PRoactive multicomponent intervention program on disability in independently living older people: a randomized controlled trial. *J Nutr Health Aging*. (2018) 22:1051–9. doi: 10.1007/s12603-018-1101-x
21. Suominen JH, Puranen TM, Jyvakorpi SK, Eloniemi-Sulkava U, Kautiainen H, Siljamaki-Ojansuu U, et al. Nutritional guidance improves nutrient intake and quality of life, and may prevent falls in aged persons with Alzheimer disease living with a spouse. *J Nutr Health Aging*. (2015) 19:901–7. doi: 10.1007/s12603-015-0558-0
22. Young AM, Mudge AM, Banks MD, Rogers L, Demedio K, Isenring E. Improving nutritional discharge planning and follow up in older medical inpatients: hospital to home outreach for malnourished elders. *Nutr Diet*. (2019) 75:283–90. doi: 10.1111/1747-0080.12408
23. Hernandez-Galiot A, Goni I. Quality of life and risk of malnutrition in a home-dwelling population over 75 years old. *Nutrition*. (2017) 35:81–6. doi: 10.1016/j.nut.2016.10.013
24. Bakker MH, Vissink A, Spoorenberg SLW, Jager-Wittenaar H, Wynia K, Visser A. Are edentulousness, oral health problems and poor health-related quality of life associated with malnutrition in community-dwelling elderly (aged 75 years and over)? A cross-sectional study. *Nutrients*. (2018) 10:1965. doi: 10.3390/nu10121965
25. Nomura Y, Kakuta E, Okada A, Otsuka R, Shimada M, Tomizawa Y, et al. Impact of the serum level of albumin and self-assessed chewing ability on mortality, QOL, and ADLs for community-dwelling older adults at the age of 85: a 15 year follow up study. *Nutrients*. (2020) 12:3315. doi: 10.3390/nu12113315
26. Gillis C, Richer L, Fenton TF, Gramilich L, Keller H, Culos-Reed N, et al. Colorectal cancer patients with malnutrition suffer poor physical and mental health before surgery. *Surgery*. (2021) 170:841–7. doi: 10.1016/j.surg.2021.04.003
27. Wham CA, Teh R, Moyes S, Dyall L, Kepa M, Hayman K, et al. Health and social factors associated with nutrition risk: results from life and living in advanced age: a cohort study in New Zealand. *J Nutr Health Aging*. (2015) 19:637–45. doi: 10.1007/s12603-015-0514-z
28. Naseer M, Fageerstrom C. Prevalence and association of undernutrition with quality of life among Swedish people aged 60 years and above: results of the SNAC-B study. *J Nutr Health Aging*. (2015) 19:970–9. doi: 10.1007/s12603-015-0656-z

29. Wham C, Curnow J, Towers A. Malnutrition risk: four year outcomes from the health, work and retirement study 2014 to 2018. *Nutrients*. 14:2205. doi: 10.3390/nu14112205
30. Maseda A, Diego-Diez C, Lorenzo-Lopez L, Lopez-Lopez R, Regueiro-Folgueira L, Milan-Calenti J. Quality of life, functional impairment and social factors as determinants of nutritional status in older adults: the VERISAÚDE study. *Clin Nutr*. (2018) 37:993–9. doi: 10.1016/j.clnu.2017.04.009
31. Toyoshima K, Araki A, Tamura Y, Ishikawa J, Kodera R, Oba K, et al. Use of dementia assessment sheet for community-based integrated care system 8-items (DASC-8) for the screening of frailty and components of comprehensive geriatric assessment. *Geriatr Gerontol Int*. (2020) 20:1157–63. doi: 10.1111/ggi.14057
32. Eriksson BG, Dey DK, Hessler RM, Steen G, Steen B. Relationship between MNA and SF-36 in a free-living elderly population aged 70 to 75. *J Nutr Health Aging*. (2005) 9:212–20.
33. Greene GW, Lofgren I, Paulin C, Greaney ML, Clark PG. Differences in psychosocial and behavioral variables by dietary screening tool risk category in older adults. *J Acad Nutr Diet*. (2018) 118:110–7. doi: 10.1016/j.jand.2017.06.365
34. Amarasena N, Keuskamp D, Balasubramanian M, Brennan DS. Health behaviours and quality of life in independently living south Australians aged 75 years or older. *Aust Dent J*. (2018) 63:156–62. doi: 10.1111/adj.12583
35. Artacho R, Lujano C, Sanchez-Vico AB, Sanchez CV, Calvo JG, Bouzas PR et al. Gonzalez J, Nutritional status in chronically-ill elderly patients. Is it related to quality of life? *J Nutr Health Aging* (2014) 18:192–197.
36. Seguy D, Hubert H, Robert J, Meunier JP, Guerin O, Raynaud-Simon A. Compliance to oral nutritional supplementation decreases the risk of hospitalisation in malnourished older adults without extra health care cost: prospective observational cohort study. *Clin Nutr*. (2020) 39:1900–7. doi: 10.1016/j.clnu.2019.08.005
37. Mori T, Nagai K, Tamaki K, Kusunoki H, Wada Y, Tsuji S, et al. Impact of quality of life on future frailty status of rural Japanese community-dwelling older adults. *Exp Gerontol*. (2022) 168:111930. doi: 10.1016/j.exger.2022.111930
